# Clinical study of the relationship between hepatitis B core antibody and mechanical ventilation in patients with Guillain-Barré syndrome

**DOI:** 10.3389/fneur.2025.1530286

**Published:** 2025-02-24

**Authors:** Wei Zhang, Qian Yao, Yuqiao Wang, Junxiong Yin, Xinxin Yang

**Affiliations:** ^1^Department of Neurology, Affiliated Brain Hospital of Nanjing Medical University, Nanjing, China; ^2^Department of Neurology, Affiliated Hospital of Xuzhou Medical University, Xuzhou, China; ^3^Dizziness Center, BenQ Medical Center Nanjing, Nanjing, China

**Keywords:** Guillain-Barré syndrome, hepatitis B virus, mechanical ventilation, predictors, risk factors

## Abstract

**Introduction:**

The aim of this study was to investigate the association between hepatitis B core antibody (HBcAb) positivity and the need of mechanical ventilation (MV) in patients with Guillain-Barré syndrome (GBS).

**Methods:**

We retrospectively analyzed the clinical data of 159 patients who were diagnosed with GBS between December 2014 and April 2023 in the Affiliated Hospital of Xuzhou Medical University. Patients were categorized into two groups according to the need for MV. Variables that were significantly different between the two groups in univariate analysis were analyzed through multivariate logistic regression models.

**Results:**

The final study population included 159 patients, 28 (17.6%) of whom need MV. In univariate analysis, Medical Research council sum score (MRC) on admission (*p* < 0.001), bulbar paralysis (*p* < 0.001), autonomic dysfunction (*p* < 0.001), HBcAb (*p* = 0.009), neutrophil/lymphocyte ratio (NLR) (*p* < 0.001), and Serum albumin (*p* = 0.016) were associated with MV. Multivariate logistic regression analysis showed lower MRC on admission (OR = 0.946, 95%CI: 0.908–0.985, *p* = 0.008), bulbar paralysis (OR = 3.726, 95%CI: 1.118–12.421, *p* = 0.032), autonomic dysfunction (OR = 3.804, 95%CI: 1.058–13.679, *p* = 0.041), HBcAb positivity (OR = 6.154, 95%CI: 1.253–30.229, *p* = 0.025), and higher NLR (OR = 1.214, 95%CI: 1.039–1.417, *p* = 0.014) were the risk factors for the need of MV in patients with GBS.

**Conclusion:**

HBcAb positivity increased the risk of MV in patients with GBS. Lower MRC on admission, bulbar paralysis, autonomic dysfunction, and higher NLR were the risk factors for the need for MV.

## 1 Introduction

Guillain-Barré syndrome (GBS) is a kind of acute inflammatory peripheral neuropathies that are mediated by the immune system (1). Even though the disease is self-limiting and immunotherapies like intravenous immunoglobulin (IVIG) and plasma exchange (PE) can help control the disease’s progression and reduce disability, a significant number of patients suffer from long-term disability or pass away from the disease (2). Respiratory complications are the main cause of death from the disease, and a recent review reported that the incidence of respiratory failure in patients with GBS ranged from 6 to 33% (3). That is why the prognosis of patients with GBS may be improved by early and precise prediction of those who will require mechanical ventilation (MV) and individualized therapy early in the course of treatment (4). The current study identified a number of factors that may be related to the need for MV in patients with GBS, such as higher neutrophil/lymphocyte ratio (NLR), bulbar paralysis, autonomic dysfunction, lower medical research council sum score (MRC) on admission, and the presence of a conduction block as demonstrated by neurophysiological assessment (5–11).

The pathomechanism of GBS is strongly correlated with prior infections, with approximately one-half to two-thirds of patients with Guillain-Barré syndrome having a history of previous infections (12). Some infectious agents that can cause GBS include *Campylobacter jejuni*, Cytomegalovirus, Epstein–Barr Virus, *Mycoplasma pneumoniae*, *Haemophilus influenzae*, Herpes simplex virus, Hepatitis B Virus (HBV), and Hepatitis E Virus (13). Japanese researchers conducted a large-scale epidemiological study showing that coexisting infectious diseases, mainly combined acute cytomegalovirus infection or herpes simplex virus, increased the risk of requiring MV in patients with GBS (14). HBV can induce the production of immune complexes or damage extrahepatic tissues through the direct viral response, resulting in various extrahepatic manifestations, with GBS being one such manifestation in the nervous system (15). GBS associated with HBV infection or HBV vaccination has been reported in numerous cases (16–18), and recurrence of GBS has been reported to be associated with reactivation or exacerbation of chronic HBV. These cases show that GBS patients who appear to be co-infected with HBV are prone to respiratory failure (19), and one study has shown that mothers infected with HBV have an increased susceptibility to respiratory disease in their offspring (20). There are no systematic studies evaluating the association between HBV infection and the need for MV in GBS patients. The hallmark antibody of HBV-infected patients is the hepatitis B core antibody (HBcAb), which is expressed during all phases of acute or chronic HBV infection (17). We conducted a retrospective study to investigate the relationship between HBcAb and the need for MV in patients who have GBS.

## 2 Materials and methods

### 2.1 Patients

In this retrospective investigation, we enrolled patients who met the diagnostic criteria for GBS (21) and had been admitted to the neurology ward of the Affiliated Hospital of Xuzhou Medical University from December 2014 to April 2023. Patients with the following features should be excluded: (1) chronic inflammatory demyelinating polyradiculoneuropathy (CIDP); (2) peripheral neuropathies caused by other etiologies, such as poisoning and diabetes mellitus; (3) the presence of neurologic illness sequelae, which complicates the determination of the severity of GBS; (4) received hormone or IVIG treatment in other hospitals; (5) Absence of five indicators of HBV or inadequate medical records. This study was approved by the Ethics Committee of the Affiliated Hospital of Xuzhou Medical University.

### 2.2 Clinical characteristics data

For all included patients, we collected data on the following characteristics: sex, age, history of chronic diseases (hypertension, diabetes, coronary artery disease, cerebrovascular disease), antecedent events (mainly upper respiratory infections and diarrhea), time from onset to admission, time from onset to nadir, MRC on admission, facial nerve palsy, bulbar paralysis, tendon reflexes, sensory deficits, autonomic dysfunction (blood pressure fluctuations, tachyarrhythmias and bradycardia, and abnormal sweating), and treatment options.

Peripheral venous blood samples collected for the first time from patients on admission to the hospital were tested for five indicators of HBV, including HBV surface antigen (HBsAg), HBV surface antibody (HBsAb), HBV e antigen (HBeAg), HBV e antibody (HBeAb), and HBcAb, as well as for neutrophils, lymphocytes, aspartate transaminase, alanine transaminase, serum albumin, uric acid, serum sodium, serum chloride, serum potassium, and NLR. Cerebrospinal fluid protein results were collected from patients who had undergone complete lumbar punctures.

Patients who had complete neurophysiology were classified into acute inflammatory demyelinating polyradiculoneuropathy (AIDP), acute motor axonal neuropathy (AMAN), acute motor sensory axonal neuropathy (AMSAN) and uncertain according to Hadden’s criteria (22). Patients not fulfilling criteria for AIDP, AMAN, and AMSAN were classified as uncertain.

### 2.3 Criteria for evaluation

The MRC is used to evaluate patients’ impaired muscle strength by examining hip flexion, knee extension, foot dorsiflexion, as well as upper arm abduction, elbow flexion, and wrist extension (a total of 5 grades of score), which are then summed up to give a total score (23). The decision to apply MV was made by the doctor in charge. Patients should meet at least one of the following major criteria or two minor criteria in order to receive MV (24). Major criteria: (1) respiratory distress; (2) hypercapnia (PaCO2 > 48 mmHg); (3) hypoxaemia (PaO_2_ < 56 mmHg); (4) spirometry ≤15 mL/kg. Minor criteria: (1) inability to cough; (2) inability to remove bronchial secretions; (3) severe bulbar dysfunction. Patients were categorized into the MV group and the non-MV group according to the need for MV.

### 2.4 Statistical analysis

SPSS version 26.0 (IBM Corp, Armonk, New York) was used for data processing and analysis. Count data are expressed as percent or ratio, which were compared using the chi-square test or Fisher exact test. The normality test was conducted using the Kolmogorov–smirnov test. Measurement data conforming to a normal distribution were expressed as mean ± standard deviation, and the *t* test was used for comparison; measurement data not conforming to a normal distribution were expressed as median and interquartile range (IQR), and the Mann–Whitney U test was used for comparison. Multivariate logistic regression was used to analyze risk factors that affect MV in patients with GBS. Furthermore, we also performed subgroup analyses to explore the relationship between HBcAb and MV in different subgroups. Two-tailed *p*-values < 0.05 were considered statistically significant.

## 3 Results

**Figure 1:**
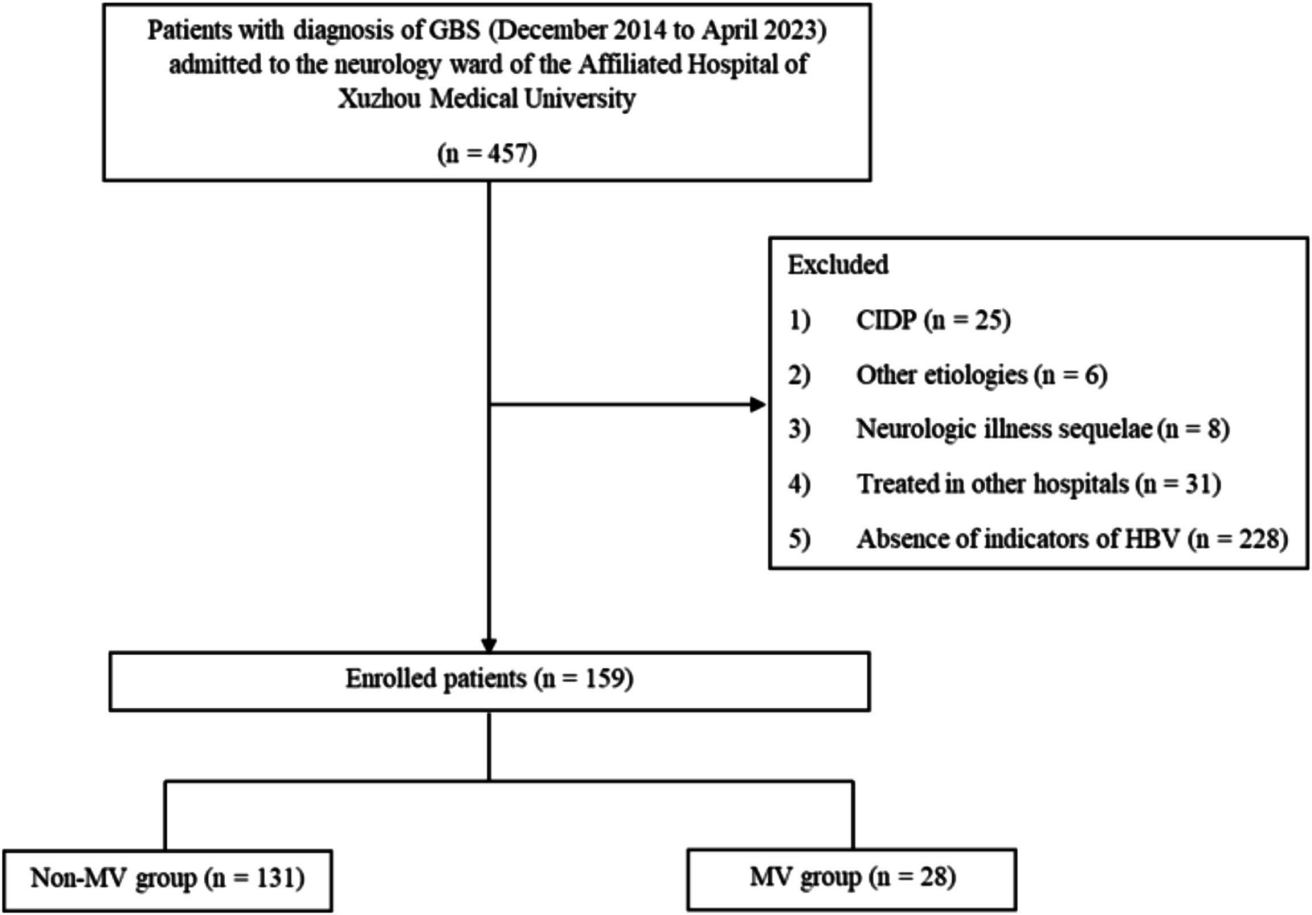
Flowchart showing the selection process of patients for the study. GBS, Guillain-Barré syndrome; CIDP, Chronic Inflammatory Demyelinating Polyneuropathy; HBV, hepatitis B virus; MV, mechanical ventilation.

A total of 159 patients with GBS were enrolled (Figure 1), the mean age of the patients was (51.07 ± 17.48) years, 96 (60.38%) patients were male patients and 63 (39.62%) patients were female patients. Electromyography was completed in 126 of 159 patients, while the remaining 33 patients were unable to cooperate or refused to complete the Electromyography. The lumbar puncture was completed in 135 of 159 patients, and protein-cell separation was present in 76.30% (103/135) of the patients, the other 24 patients were unable to cooperate or refused to perfect lumbar puncture. The most common treatment was IVIG, which was applied in 146 (91.82%) patients, plasma exchange (PE) was applied in 3 (1.89%) patients, and both IVIG and PE were applied in 4 (2.52%) patients. For financial or allergy-related reasons, 10 (6.29%) patients received just general symptomatic treatment or hormone. The MV group included 28 patients (17.61%) who had respiratory failure requiring MV, the remaining 131 patients were included in the non-MV group.

Compared with the non-MV group, patients in the MV group had more common bulbar paralysis, autonomic dysfunction, HBcAb positivity, higher NLR, but lower MRC and serum albumin, while the rest of the indices were not statistically different between the two groups (Table 1).

**Table 1:** Clinical characteristics of patients with GBS: Comparison of MV group and non-MV group.

Variables	Total (*n* = 159)	Non-MV (*n* = 131)	MV (*n* = 28)	*p*-value
Age (years), mean ± SD	51.1 ± 17.5	50.1 ± 17.1	55.6 ± 18.7	0.128
Male gender (%)	96 (60.4%)	75 (57.3%)	21 (75.0%)	0.081
History of chronic diseases (%)	63 (39.6%)	49 (37.4%)	14 (50.0%)	0.216
Antecedent events (%)	106 (66.7%)	88 (67.2%)	18 (64.3%)	0.768
Time from onset to admission (days), median (IQR)	4.0 (3.0, 7.0)	4.0 (3.0, 7.0)	3.5 (2.0, 6.0)	0.159
Time from onset to nadir (days), median (IQR)	5.0 (3.0, 7.5)	5.0 (3.0, 7.0)	5.5 (2.0, 8.0)	0.795
MRC on admission (score), median (IQR)	44.0 (31.0, 50.0)	46.0 (36.0, 52.0)	24.0 (12.0, 36.0)	*p* < 0.001
Facial nerve palsy (%)	40 (25.2%)	32 (24.4%)	8 (28.6%)	0.646
Bulbar paralysis (%)	55 (34.6%)	35 (26.7%)	20 (71.4%)	*p* < 0.001
Hyporeflexia (%)	140 (88.1%)	113 (86.3%)	27 (96.4%)	0.236
Sensory deficits (%)	94 (59.1%)	79 (60.3%)	15 (53.6%)	0.511
Autonomic dysfunction (%)	61 (38.4%)	39 (29.8%)	22 (78.6%)	*p* < 0.001
**Electrophysiology**				0.286
AIDP (%)	80 (63.5%)	72 (64.3%)	8 (57.1%)	
AMAN (%)	22 (17.5%)	20 (17.9%)	2 (14.3%)	
AMSAN (%)	10 (7.9%)	7 (6.3%)	3 (21.4%)	
Uncertain (%)	14 (11.1%)	13 (11.6%)	1 (7.1%)	
HBcAb positivity (%)	96 (60.4%)	73 (55.7%)	23 (82.1%)	0.009
NLR, median (IQR)	3.3 (2.3, 5.6)	2.7 (2.2, 4.3)	8.3 (4.8, 15.3)	*p* < 0.001
Aspartate transaminase (u/L), median (IQR)	22.0 (18.0, 30.5)	22.0 (18.0, 29.0)	25.5 (19.3, 35.3)	0.132
Alanine transaminase (u/L), median (IQR)	24.0 (16.5, 38.5)	24.0 (16.0, 39.0)	26.0 (19.0, 37.0)	0.552
Serum albumin (g/L), mean ± SD	41.7 ± 5.5	42.3 ± 4.9	38.7 ± 7.2	0.016
Uric acid (umol/L), mean ± SD	252.7 ± 90.7	250.4 ± 84.4	263.7 ± 117.2	0.482
Serum sodium (mmol/L), median (IQR)	139.4 (136.9, 141.7)	139.7 (137.0, 141.8)	138.3 (136.7, 139.9)	0.082
Serum chloride (mmol/L), median (IQR)	101.4 (98.7, 103.9)	101.5 (98.7, 103.9)	100.7 (98.5, 103.1)	0.513
Serum potassium (mmol/L), median (IQR)	4.0 (3.8, 4.3)	4.0 (3.8, 4.3)	4.0 (3.7, 4.4)	0.750
Protein-cell separation (%)	103 (76.3%)	87 (77.0%)	16 (72.7%)	0.667
Cerebrospinal fluid protein (g/L), median (IQR)	0.8 (0.5, 1.3)	0.8 (0.5, 1.3)	0.8 (0.5, 1.1)	0.793
IVIG or PE (%)	149 (93.7%)	122 (93.1%)	27 (96.4%)	0.823

MV, mechanical ventilation; MRC, Medical Research council sum score; AIDP, acute inflammatory demyelinating polyradiculoneuropathy; AMAN, acute motor axonal neuropathy; AMSAN, acute motorsensory axonal neuropathy; HBcAb, hepatitis B core antibody; NLR, neutrophil/lymphocyte ratio; IVIG, intravenous immunoglobulin; PE, plasma exchange.

**Figure 2:**
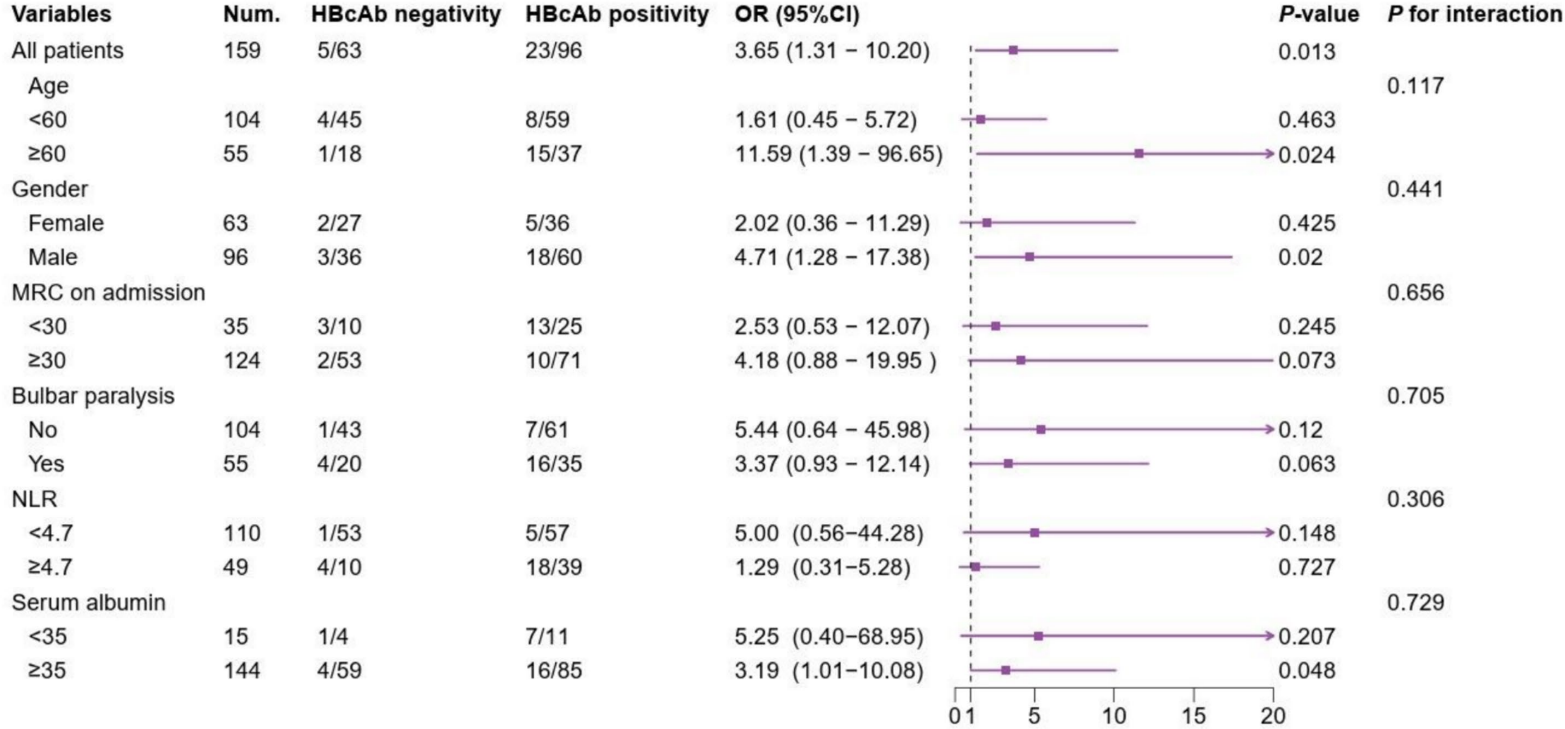
Subgroup analyses of the association between HBcAb and MV in patients with GBS. NUM, number; HBcAb, hepatitis B core antibody; MRC, Medical Research council sum score; NLR, neutrophil/lymphocyte ratio.

The multivariate logistic regression analysis comprised indicators that showed statistically significant differences in the univariate analysis (Table 1). Multivariate logistic analysis revealed that lower MRC on admission, bulbar paralysis, autonomic dysfunction, HBcAb positivity, and higher NLR were the risk factors for the need of MV in patients with GBS (Table 2). The correlation between HBcAb and MV remained consistent across various subgroups, unaffected by factors such as age, gender, MRC on admission, bulbar paralysis, NLR, and serum albumin (all *P* for interaction > 0.05) (Figure 2).

**Table 2:** Multivariate logistic regression analysis of risk factors for MV in patients with GBS.

Variables	β	SE	Wald	*p*-value	OR	95%CI
MRC on admission	−0.056	0.021	7.084	0.008	0.946	0.908–0.985
Bulbar paralysis	1.315	0.614	4.585	0.032	3.726	1.118–12.421
Autonomic dysfunction	1.336	0.653	4.186	0.041	3.804	1.058–13.679
HBcAb positivity	1.817	0.812	5.006	0.025	6.154	1.253–30.229
NLR	0.194	0.079	5.984	0.014	1.214	1.039–1.417

MRC, Medical Research council sum score; HBcAb, hepatitis B core antibody; NLR, neutrophil/lymphocyte ratio.

## 4 Discussion

GBS is a self-limiting disease, the majority of patients can fully recover, but respiratory failure requiring MV is a serious complication of GBS. According to previous studies, the incidence of respiratory failure in patients with GBS ranges from 6 to 33% (3). The current study found that 17.61% of patients with GBS had respiratory failure, falling within this range. Multivariate logistic regression analysis in this study revealed that HBcAb positivity, low MRC on admission, bulbar paralysis, autonomic dysfunction, and higher NLR were risk factors for the requirement for MV in patients with GBS.

The major HBV serological markers include HBsAg, HBsAb, HBeAg, HBeAb, HBcAb. During different stages of HBV infection, the other four markers may be falsely negative, leading to the missed diagnosis of patients with HBV infection, while HBcAb is the hallmark antibody of HBV infection, which is expressed in the serum of all kinds of HBV-infected patients (25), hence, HBcAb positivity was selected as an observational marker of HBV infection in patients with GBS in this investigation. HBV infection induces the production and deposition of immune complexes, induced autoantibodies reacting with tissue antigens, or the presence of direct viral reactions, which may occur in extrahepatic tissues such as the nervous system, skin, muscles, joints, and kidneys. GBS is a rare neurologic complication of HBV infection (17). The definitive mechanism by which HBV induces GBS is unclear, but on the basis of published studies to date, there are three possible mechanisms. The first is immune complex damage caused by the virus; in prior case reports of GBS with HBV infection, immunofluorescence was used to detect the presence of hepatitis B virus surface antigen-positive markers on the nerve endothelium and small vessels of the nerve endothelium of patients with GBS (26). Serum and cerebrospinal fluid titers of hepatitis B virus surface antigen immune complexes increase in patients with GBS during the acute phase; these titers decrease following antiviral treatment and the recovery of neurologic function (26, 27). These findings suggest that HBV is likely to induce GBS through the formation of immune complexes leading to peripheral neurovasculitis and eventually to GBS. The second is direct viral damage; patients with GBS have HBV DNA found in their cerebrospinal fluid, thus, it is proposed that direct infection of the peripheral nerve system by the HBV may result in Guillain-Barré syndrome (28). The third is an indirect immune response known as “molecular mimicry,” in which the HBV infection may cause the production of antibodies and the activation of monocytes through the amino acid sequences of the HBV polymerase mimicking one of the polypeptides in the basic protein of the peripheral nerve myelin sheath (29). Studies on *Campylobacter jejuni* and Zika virus infections have shown this process, which allows patients with infection-related GBS to produce autoimmune antibodies through molecular mimicry that are specific to GBS, particularly ganglioside antibodies (1). When T cells and macrophages recognize cross-reactive antigens, B cells respond with an anti-ganglioside, which breaks down the blood–brain barrier, activates complement, activates macrophages in the nerve endothelium, and releases cytokines and free radicals (such as nitric oxide), which can cause axonal damage or loss of the myelin sheath. Activated T lymphocytes harm Schwann cells and nerve terminals simultaneously by activating complement and releasing pro-inflammatory cytokines (30).

HBV can cause lung damage in patients with infection by numerous mechanisms, including immune complex formation. The virus’s surface antigens can produce polyarteritis nodosa, when the virus deposits itself in the blood vessels of multiple organs. Likewise, these immune complexes have the ability to accumulate in the lungs’ tiny blood arteries and capillaries, which impedes gas exchange and results in obstructive ventilation (31). Immune complexes mediated by cryoglobulins are also deposited in the vascular and interstitial tissues of the lungs, which may be a possible mechanism contributing to lung dysfunction (20). Furthermore, HBV activates the release of inflammatory agents that disrupt the physiologic healing response to lung injury, thus making the lungs susceptible to injurious factors (32). Our study demonstrated for the first time by retrospective analysis that HBV infection is a risk factor for the need for mechanical ventilation in patients with GBS, and we speculate that this is due to the fact that patients infected with HBV are more likely to develop respiratory failure because their lung function is already impaired prior to the onset of GBS as compared to uninfected patients.

Some of the previously reported risk factors were also demonstrated in our study. We used the MRC to evaluate the degree of muscle weakness on admission, which shows that lower MRC on admission is a risk factor for the need for MV, meaning that the more severe the paralysis on admission, the higher the probability of needing MV. This might be since patients who have severe muscular weakness on admission tend to be bedridden longer, which increases their risk of suffering from respiratory conditions such aspiration pneumonia and eventual respiratory failure (3). Bulbar paralysis is characterized by dysphagia and dysarthria, which can lead to sputum inability to be coughed up, aspiration pneumonia, and even asphyxia, inhibiting the patient’s respiratory function (33). The autonomic dysfunction that occurs in many patients with GBS (34), which decreases airway vagal tone and the ventilatory response to hypoxia or hypercapnia, promotes the accumulation of respiratory mucus plugs (35), leading to respiratory failure and death in patients with GBS (36). The NLR is an effective indicator of the systemic inflammatory response and a rapid-responding biomarker of cellular immune activation. In GBS patients, the higher NLR is indicative of an underlying pro-inflammatory condition and immune system dysregulation (37). Neutrophils are central components of the innate immune system that enhance pro-inflammatory immune responses to fight pathogens and clear foreign substances from the body (38). Lymphocytes, which are central players in the adaptive immune system, attenuate pro-inflammatory responses and modulate immune responses. Patients with chronic respiratory failure have a higher NLR than healthy individuals (39), and the NLR has been recognized as a risk factor for the poor prognosis of ventilator-associated pneumonia (40). In addition, it has been argued that a shorter time from onset to admission or time from onset to nadir is a risk factor for the need for MV in patients with GBS (9). However, our study did not show the correlation, which may be related to the fact that the patients with GBS who were included in the analysis came to the hospital when their symptoms were minor and received treatment with IVIG earlier in our hospital.

There are several limitations to this study. First, HBcAb positivity represents the existence of HBV infection, but it does not indicate that HBV infection is in acute exacerbation, chronic exacerbation, or only occult infection; hence, quantitative HBV DNA testing is needed to determine the presence of viral replication in patients with GBS who have HBcAb positivity; but this study was a retrospective analysis and unable to quantitative HBV DNA testing was obtained. Second, because the study was retrospective and some patients were too severe to cooperate with the examination, not all the enrolled patients were perfected with the two examinations of electromyography and lumbar puncture, which might affect our comparative analysis of neurophysiology and cerebrospinal fluid. Thirdly, this study is based on a single center population with a small number of cases, which needs to be further expanded for in-depth study.

In conclusion, HBcAb positivity increased the risk of MV in patients with GBS. Lower MRC on admission, bulbar paralysis, autonomic dysfunction, and higher NLR were the risk factors for the need for MV.

## Data Availability

The raw data supporting the conclusions of this article will be made available by the authors, without undue reservation.
